# Desflurane for management of decompressive laminectomy in a patient with hereditary spastic paraplegia: a case report

**DOI:** 10.1186/s40981-019-0250-1

**Published:** 2019-04-30

**Authors:** Masahiro Tada, Kazuyoshi Tateoka, Kenji Yamamoto, Yasuyoshi Inagaki, Takayuki Kunisawa

**Affiliations:** 1 Department of Anesthesiology and Critical Care Medicine, Asahikawa Medical University, Midorigaoka-higashi 2-1-1-1, Asahikawa, Hokkaido 078-8510 Japan; 2 Department of Anesthesiology, National Hospital Organization Obihiro Hospital, Obihiro, Hokkaido Japan; 3 Department of Anesthesia, Nayoro City General Hospital, Nayoro, Hokkaido Japan; 4 Department of Emergency Medicine, Nayoro City General Hospital, Nayoro, Hokkaido Japan

**Keywords:** Hereditary spastic paraplegia, Desflurane, General anesthesia, Rocuronium, Sugammadex

## Abstract

**Background:**

Hereditary spastic paraplegia (HSP) is a rare, genetic neurodegenerative condition. Thus far, ideal anesthetic management is not established for patients with HSP; therefore, careful selection and dosage of anesthetic agents is required.

**Case presentation:**

A 54-year-old woman with HSP, who was diagnosed with severe lumbar spinal canal stenosis, underwent decompressive laminectomy to relieve her back pain. Preoperatively, she experienced slight difficulty in walking independently; however, she exhibited no other dysfunction. Anesthesia was maintained with desflurane after tracheal intubation. Rocuronium and sugammadex were used for neuromuscular blockade and reversal, respectively, with neuromuscular monitoring equipment. The patient showed uneventful postoperative recovery without signs of neurological deterioration after extubation.

**Conclusions:**

Our successful experience in this case implies that, for patients with neuromuscular diseases, including HSP, desflurane may be an option for anesthetic management; moreover, careful assessment (e.g., medical condition, bispectral index, and train-of-four) should be performed prior to administration of anesthesia.

## Background

Hereditary spastic paraplegia (HSP) comprises a group of heterogeneous neurodegenerative diseases, characterized by genetic mutations that cause distal neuropathy of the longest corticospinal tract axons [1, 2]. Thus far, 55 spastic paraplegia genes (SPGs) have been identified. Mutations in specific genes, including SPG3A/atlastin, SPG4/spastin, and SPG7/paraplegin, cause distinct types of HSP (SPG3A, SPG4, and SPG7 HSP, respectively) [2, 3]. In this pathophysiologic condition, primary clinical features are progressive lower limb weakness and spasticity [4].

Notably, HSP is a rare disease, estimated to affect 1 to 10 individuals per 100,000 individuals in Europe [5–7], and approximately 0.2 individuals per 100,000 individuals in Japan [8]. A few reports [4, 5, 9, 10] have described general anesthetic management for patients with HSP; these reports revealed the use of sevoflurane (with or without nitrous oxide) or propofol as anesthetic agents. The ideal anesthetic agent has not been established for general anesthesia for patients with HSP. In contrast to the previously mentioned anesthetics, desflurane can be a useful anesthetic agent for spinal surgery in patients with HSP because of its characteristics, such as rapid recovery [11–13] and muscle relaxation [11].

Here, we describe the successful use of desflurane for general anesthesia during decompressive laminectomy in a female patient with HSP, in accordance with her informed consent for publication of the anonymized case details and images.

## Case presentation

A 54-year-old woman (height 156 cm, weight 48 kg) with a 15-year history of HSP was diagnosed with lumbar spinal canal stenosis. Her chief complaint was lower back pain upon standing. Her symptoms showed progressive worsening, with severe stenosis at L4/5; consequently, decompressive laminectomy was scheduled to relieve the pain. Neurological examination revealed respective left and right straight-leg raise angles of 50° and 50°, bilateral patellar tendon reflexes were exaggerated, bilateral Achilles tendon reflexes were absent, and left and right manual muscle testing showed (5, 5; 2, 2; 3, 3; and 3, 3 [left and right, respectively]) at the tibialis anterior, lower limb extensor group, lower limb flexor, and gastrocnemius muscles, respectively. Although the patient experienced slight difficulty in standing and walking, she exhibited no dysfunction in the upper limbs; moreover, she showed no bladder or rectal disturbances. Tactile sensory function was intact. She began rehabilitation, comprising standing and walking training to achieve stable walking. She received intrathecal baclofen therapy (38 mcg/day), which relieved the symptom of spasticity.

The patient had undergone laparoscopic unilateral salpingo-oophorectomy using general anesthesia with propofol, remifentanil, and rocuronium, 2 years prior to the present operation. There were no adverse events, such as malignant hyperthermia. Preoperative examinations during the current surgery (e.g., electrocardiography, spirometry, laboratory data, and chest radiography) were unremarkable. The Mallampati classification was I, and the degree of mouth opening (i.e., initial interincisal distance) was > 35 mm. The patient had no smoking history. The patient presented to the hospital 1 day prior to surgery. She was permitted to move using a wheelchair and had no other limitations with regard to her behavior in the ward.

To measure the train-of-four (TOF), TOF-Watch (NIHON KOHDEN Corporation, Tokyo, Japan) was used as a peripheral nerve stimulator; neuromuscular activity was monitored at the adductor pollicis muscle in response to ulnar nerve stimulation. Before induction of general anesthesia, the left radial artery was catheterized for arterial line and blood gas assessment (BGA), which revealed a partial pressure of arterial carbon dioxide (PaCO_2_) of 36.9 mmHg and a partial pressure of arterial oxygen (PaO_2_) of 82.6 mmHg.

After pre-oxygenation, general anesthesia was induced using propofol (60 mg [1.2 mg/kg]) and remifentanil (0.3 mcg/kg/min) for rapid induction; rocuronium (10 mg) was then infused. When the TOF ratio decreased and TOF counts reached 4 (2 min after initial rocuronium infusion), additional rocuronium (10 mg) was added (a total of 20 mg rocuronium was administered). TOF counts reached 0 after additional rocuronium administration. Tracheal tube intubation was performed easily using a videolaryngoscope (Airway Scope, AWS-S100, HOYA, Tokyo, Japan). Anesthesia was maintained using desflurane (end-tidal concentration 4.0–4.2%) in oxygen and air (fraction of inspired oxygen 0.4) and remifentanil (0.1–0.4 mcg/kg/min). Fentanyl (50 mcg/dose or 100 mcg/dose) was infused (total of 250 mcg, i.e., 5 mcg/kg was administered). Rocuronium (10 mg) was infused (0.2 mg/kg) when TOF count reached 1. The total dosage of rocuronium was 40 mg in 2.5 h. Vital signs and bispectral index (approximately 40) were stable intraoperatively. In total, 1.4 L of crystalloid was infused intraoperatively. Sufficient akinesia was achieved, and surgery was completed safely.

Neuromuscular blockade was reversed using sugammadex 100 mg (2 mg/kg); postoperative TOF counts were 4. A TOF ratio > 0.9 was obtained. The patient uneventfully emerged from anesthesia. She was rapidly able to follow instructions and breathe spontaneously while intubated and demonstrated adequate tidal volumes before extubation.

After extubation, oxygen saturation remained > 99% when supplying oxygen at 3 L/min. Her respiratory rate was 16 breaths/min and PaCO_2_ and PaO_2_ were 46.4 mmHg and 150 mmHg, respectively. Other parameters were approximately identical to their preoperative measurements. The patient’s respiratory condition was stable, without signs of neurological deterioration.

The patient postoperatively used NSAIDs when she experienced wound pain or headache without opioids. One week postoperatively, the patient exhibited fever, which led to a diagnosis of wound infection. She underwent curettage, and the wound was washed and drained. Antibiotics were used to enable her to recover from the wound infection. The patient was recommended bed rest postoperatively; she was permitted to reposition to Fowler’s position if there was no pain. On postoperative day 12, the drain tube was removed, and she was allowed to move using a wheelchair. The patient restarted rehabilitation on postoperative day 18. She trained by standing and walking and gradually recovered to her preoperative level of mobility. She was finally discharged home on postoperative day 43.

## Discussion

Our experience in this case indicates that, for patients with HSP, desflurane can be successfully used to maintain general anesthesia during decompressive laminectomy, in combination with commonly used agents (propofol and remifentanil). Earlier studies reported the use of sevoflurane [4, 9, 10] and propofol [5] for the management of various surgeries in patients with HSP.

HSP is associated with multiple difficulties in anesthetic management because it involves neurodegenerative disease; therefore, it is important to correctly establish the pathophysiology of affected patients. First, perioperative respiratory complications can occur; such complications include pulmonary infections. Patients with neurological diseases commonly exhibit poor respiratory muscle function, with associated reduction of respiratory reserve. Second, administration of depolarizing muscle relaxants, such as suxamethonium, can cause hyperkalemia. Third, such patients may be hypersensitive to non-depolarizing muscle relaxants [14]; therefore, normal or slightly increased doses may be sufficient for intubation. In this case, the patient exhibited no respiratory dysfunction. Indeed, preoperative spirometry testing and BGA remained intact. Postoperatively, the patient’s respiratory muscles sufficiently recovered from the muscle relaxant; this was reflected in clinical signs, neuromuscular monitoring, and postoperative BGA. She experienced no pulmonary infections postoperatively.

Compared to suxamethonium, which may cause hyperkalemia, rocuronium is considered appropriate for intubation in neurodegenerative patients [10]. However, patients with HSP may exhibit altered sensitivity to rocuronium, compared to non-HSP patients [10]; therefore, the effect of muscle relaxants should be monitored. Notably, peripheral nerve stimulation is reportedly an unreliable guide for relaxant dosages in patients with upper motor neuron lesions [15]; nevertheless, it is necessary to use muscle relaxant carefully and to closely monitor clinical states and peripheral nerve stimulation results.

Classification of neurodegenerative disorders, including HSP, is a complicated process and can mask other diseases; however, genes responsible for some neurodegenerative diseases have been detected recently [16]. For safe anesthetic management, adequate assessment of the patient’s disease is needed, in order to correctly judge the risks and benefits of each anesthetic strategy.

Thus far, specific problems concerning anesthetic management for patients with HSP, relative to other motor neuron diseases, have not been established in the literature; however, Ponsonnard et al. [5] noted that HSP comprised a heterogeneous group of genetic diseases, such that there was no certainty that anesthetic drugs would have the same action among all variants of HSP. Careful perioperative monitoring and assessment of the effect of anesthetic drugs are needed. For this purpose, desflurane with bispectral index enables simple anesthetic management because the target expiratory concentration can be adjusted on the basis of its solubility [11]. In this case, the patient exhibited no dysfunction, except in the lower limbs. We believed there was a low risk involved in performing general anesthesia with intubation using desflurane with careful monitoring; there were no perioperative problems.

Here, we have described the successful use of desflurane for general anesthesia to manage complications associated with neurological conditions during decompressive laminectomy in a patient with HSP. We suggest that desflurane, in combination with other routinely used and established anesthetics, is a satisfactory anesthetic agent for such patients. Importantly, desflurane may be an option for safe and effective anesthetic management if the patient’s disease condition and anesthetic states are closely and carefully monitored.

## Abbreviations

BGA: Blood gas assessment
HSP: Hereditary spastic paraplegia
PaCO_2_: Partial pressure of arterial carbon dioxide
PaO_2_: Partial pressure of arterial oxygen
TOF: Train-of-four
